# 3D Bite Modeling and Feeding Mechanics of the Largest Living Amphibian, the Chinese Giant Salamander *Andrias davidianus* (Amphibia:Urodela)

**DOI:** 10.1371/journal.pone.0121885

**Published:** 2015-04-08

**Authors:** Josep Fortuny, Jordi Marcé-Nogué, Egon Heiss, Montserrat Sanchez, Lluis Gil, Àngel Galobart

**Affiliations:** 1 Institut Català de Paleontologia Miquel Crusafont, Sabadell, Spain; 2 Universitat Politècnica de Catalunya—BarcelonaTech, Terrassa, Spain; 3 Department of Integrative Zoology, University of Vienna, Vienna, Austria; 4 Institute of Systematic Zoology and Evolutionary Biology, Friedrich-Schiller-University Jena, Jena, Germany; University of Liverpool, UNITED KINGDOM

## Abstract

Biting is an integral feature of the feeding mechanism for aquatic and terrestrial salamanders to capture, fix or immobilize elusive or struggling prey. However, little information is available on how it works and the functional implications of this biting system in amphibians although such approaches might be essential to understand feeding systems performed by early tetrapods. Herein, the skull biomechanics of the Chinese giant salamander, *Andrias davidianus* is investigated using 3D finite element analysis. The results reveal that the prey contact position is crucial for the structural performance of the skull, which is probably related to the lack of a bony bridge between the posterior end of the maxilla and the anterior quadrato-squamosal region. Giant salamanders perform asymmetrical strikes. These strikes are unusual and specialized behavior but might indeed be beneficial in such sit-and-wait or ambush-predators to capture laterally approaching prey. However, once captured by an asymmetrical strike, large, elusive and struggling prey have to be brought to the anterior jaw region to be subdued by a strong bite. Given their basal position within extant salamanders and their “conservative” morphology, cryptobranchids may be useful models to reconstruct the feeding ecology and biomechanics of different members of early tetrapods and amphibians, with similar osteological and myological constraints.

## Introduction

The uptake of food is vital for all animal life, and feeding adaptations suited to specific environments in order to exploit food sources were essential for vertebrate evolution [1–2]. The wide range of lifestyles and trophic ecologies exhibited in salamanders has led to diverse feeding systems. Aquatic salamanders typically capture their prey by suction feeding, where a fast oropharyngeal volume expansion pulls prey and surrounding water into the gaping mouth [3–8]. Action of the jaws usually then immobilize evasive or struggling prey. By contrast, terrestrial feeding involves capturing prey directly by the jaws or, in more specialized forms, capturing prey by the quickly protracted and retracted tongue [6,8–11]. Analogous to aquatic prey capture, the jaws and dentition are then used for fixing and immobilizing the prey. Accordingly, the biting apparatus is essential for successful prey capture and processing both in aquatic and terrestrial strikes and the prey spectrum is constrained by the functional architecture of the jaw-skull system, which has to cope with different levels of stress [5,11]. Former studies have shown that biomechanical approaches involving the analyses of movements and forces, combined with theoretical models, can help explain the divergence of the feeding systems in animals occupying different ecological niches [12].

Biting mechanisms in salamanders are essential tools to capture, fix, subdue and kill prey both on land and in water [5, 11]. However, limited information is available in the literature about how it works and the functional implications of biting system in amphibians in general and in salamanders in particular are still poorly understood although such knowledge might be essential to understand feeding systems performed by early tetrapods.

Computational methods from mechanics and structural analysis-such as finite element analysis (FEA) or multibody dynamics analysis (MDA) provide powerful tools to explore the biomechanical performance of the skull. Multibody dynamics analysis (MDA) is the study of the dynamic behavior of interconnected rigid or flexible bodies, each of which may undergo large translational and rotational displacements. Using MDA is possible to simulate rigid-body motion of the external forces and internal musculature responsible for skull loading [13]. FEA [14, 15] has been conducted in a wide diversity of vertebrates providing new insights to explore the function, morphological evolution, particular adaptation and constraints of the biological structures, as skulls [14]. Previous works analyzed the morphological evolution of the skull on early tetrapods using simplified 2D models with the goal to analyze the function and the biomechanical capabilities of the largest group of early tetrapods, the Temnospondyls [16–17]. FEA is especially useful to investigate the stress patterns of the skull in animals that are difficult or impossible to study in vivo (e.g. dinosaurs, small animals) [14–15,18]. FEA also allows us to investigate the stress during different feeding regimes such as unilateral, bilateral, and anterior versus posterior bite points without invasive techniques [14].

Herein, we investigate the skull biomechanics during a bite in the Chinese giant salamander, *Andrias davidianus* using 3D finite element analysis (FEA). This taxon is a member of the basal urodele clade Cryptobranchidae. Giant salamanders are of special interest from an evolutionary point of view as they belong to the oldest extant amphibian groups with an origin dating from the Middle Jurassic (161 Myr) sharing many ancestral characteristics [19–22]. In fact, early amphibians were characterized by large, salamander-like aquatic predators with large, flat and broad skulls [21], similar to the extant giant salamanders, and accordingly, extant cryptobranchids might be useful models to deduce the function of early amphibian feeding systems [7].

Accordingly, the aims of the study are to: a) obtain the stress distribution of this taxon under different loadings in a computational model; b) test models of asymmetrical feeding in *A*. *davidianus* and discuss the implications; c) evaluate the significance of the morphological changes on the feeding apparatus during growth in this taxon; d) explore the potential evolutionary implications for stem-amphibians that gave rise to extant lissamphibians.

## Material and Methods

### The sample

The genus *Andrias* is represented by two living species of giant salamanders and includes the largest extant amphibians. The Chinese giant salamander, *Andrias davidianus* is the largest living amphibian reaching a total length (TL) up to 1.5 meters. The analyses were conducted on two skulls from one adult and one subadult. The adult specimen (stock no. 1/2009) 127 cm TL, is stored at the Zoological Collection of the Department of Theoretical Biology, University of Vienna, Austria. The skull length from the tip of the premaxilla to the occipital condyle is 18 cm. The subadult specimen (MZB–2001-0961-B) is stored at the Museu de Ciències Naturals de Barcelona (Catalonia), total length of the specimen is unknown, had a skull length of 9 cm from the tip of premaxilla to the occipital condyles.

### Computed Tomography

The adult specimen was scanned with a Somatom emotion medical multi-slice CT scanner (Siemens AG, Germany) at the Clinic of Diagnostic Imaging University of Veterinary Medicine Vienna (Austria) using 130 kV, 100 mA, obtaining 0.725 mm of pixel size and an output of 512 x 512 pixels per slice with an interslice space of 0.4 mm. The subadult specimen was scanned with a Sensations-16 CT scanner (Siemens AG, Germany) at Hospital Mútua de Terrassa (Barcelona, Catalonia) using 140 kV, 150 mA, obtaining 0.586 mm of pixel size and an output of 512 x 512 pixels per slice with an interslice space of 0.1 mm.

### Geometry reconstruction

The CT data was imported to the software Avizo 7.0 (VSG, Germany), where the three-dimensional reconstruction and segmentation was performed. Cranial sutures were not included in the FE model but were described and discussed in the light of the FEA results to understand their mechanical significance.

-The 3D models were then converted to a CAD model using reverse engineering techniques [23] which is the process of discovering the technological principles of a device, object or system through analysis of its structure, function or operation. In the studied case, the reverse engineering procedure was used to understand the biological structures of *Andrias davidianus*.

During the reconstruction process, irregularities in the surface that are due to the generation of the model from the CT scanner were repaired using refinement and smoothing tools from Rhinoceros 5.0 software.

### Model properties

A Structural Static Analysis was performed using the Finite Element Package ANSYS 14.5 in a Dell Precision Workstation T7600 with 32 GB (4X8GB) and 1600 MHz to evaluate the role of the adductor musculature complex (see below).

The cranial bone properties for living salamanders or any other anamniote are unkown. Elastic, linear and homogeneous material properties were assumed for the bone of the models, using the following values based on the frontal and prefrontal bones from *Crocodylus*: E (Young’s modulus): 6.65 GPa and v (Poisson’s ratio) 0.35 [24]. Potential orthotropic properties of salamander skull bone were also not included. It was also not possible to obtain realistic displacements when using estimated values of material properties (E and v), but they provided a reasonable qualitative estimation of the bone performance and, more importantly, the stress distribution on the skull model should not vary under different material properties. This is because the equations of the elasticity used in FEA for linear isotropic and homogeneous materials do not affect stress patterns. The stress patterns depend on the direction and values of the forces applied because it meets the equilibrium equation between external forces and internal forces [25].

In spite of this, the use of the same linear and homogeneous material properties in a comparative analysis for different specimens have been demonstrated to be useful for studying biological questions [14] and, according to the sensitivity analysis of [26], results for heterogeneous properties of the bone in the model closely match the results for homogeneous properties (See [27] for further discussion). The skulls were meshed with an adaptive mesh of 10-noded tetrahedral elements [28]. An adaptive mesh is a type of mesh in which areas of complex geometry and areas of expected high gradients of stress have greater element density than less complex areas [14] to assure a good interpolation of the variations in the stress patterns. The mesh of the adult specimen was approximately 1.8 milion elements and 2.6 million nodes whereas the mesh of the subadult specimen was approximately 1.2 milion elements and 1.7 milions nodes. See S1 Fig.

### Boundary and loading conditions

The adductor musculature was modelled by inclusion of the adductor mandibulae externus (AME) and the adductor mandibulae internus (AMI) (.). The origin and insertion of the AME and AMI in salamanders has been described in the literature [7, 29–35]. According to previous anatomical studies [7, 29, 31, 36] the AME in *Andrias* originates from the anterior part of the squamosal, runs ventrally and inserts on the coronoid part of the mandible, as well as lateral and ventral to it on the dentary. The AMI has a more complex arrangement. It originates from the fasciae of the epaxialis musculature and more anteriorly from the parietal, frontal, and nasal region of the skull roof. Its fibers run ventrally to insert slightly anterior and medial to the insertion site of the AME on the mandible [7, 29, 31, 36]. The adductor mandibulae posterior (AMP) is small and usually poorly differentiated muscle in salamanders [32] and was not considered in this study.

The areas of muscular insertion of AMI and AME were defined in the geometric models in order to apply the forces of the muscular contraction during the prehension. The direction of these forces was defined by the line that joins the centroid of the insertion area in the skull with its insertion location on the jaw. A gape angle of 6° describing the elevation of the skull was assumed. Additionally, a maximum gape angle of 21° was also tested considering 6° of elevation of the skull and a depression of the mandible of 15° as reported *in vivo* specimens [7].

In the adult specimen, a value of 0.3 MPa (force per unit area) was assumed as muscular contraction pressure in AMI and AME according to the value given from [37]. A constant PCSA was assumed for the muscles. It allowed us to apply the muscle pressure directly into the area of its muscular insertion (Tables 1 and 2).

**Table 1:** Muscle forces applied under volume scaled cases.

**Case**	**Specimen**	**Body Volume [mm** ^3^ **]**	**AME Area [mm** ^2^ **]**	**AME Pressure [MPa]**	**AME Force [N]**	**AMI Area [mm** ^2^ **]**	**AMI Pressure [MPa]**	**AMI Force [N]**
A, B, C, D	Adult *A*. *davidianus*	144340	559.23	0.3	167.77	2333	0.3	670
E, F, G, H	Subadult *A*. *davidianus*	24964	122.11	0.42	52.08	629.05	0.345	217.26

**Table 2:** Muscle forces applied under area scaled cases.

**Case**	**Specimen**	**Body Area [mm** ^2^ **]**	**AME Area [mm** ^2^ **]**	**AME Pressure [MPa]**	**AME Force [N]**	**AMI Area [mm** ^2^ **]**	**AMI Pressure [MPa]**	**AMI Force [N]**
A, B, C, D	Adult *A*. *davidianus*	72323	559.23	0.3	167.77	2333	0.3	670
E, F, G, H	Subadult *A*. *davidianus*	16166	122.11	0.307	37.5	629.05	0.248	156.44

To allow the comparison of the results obtained in the adult and subadult specimens, as the two skulls differ in shape and size, the values of muscular contraction pressure for the subadult specimen were calculated following the theoretical methodology previously developed [38] (see below more details about the scaling procedure).

**Fig 1:**
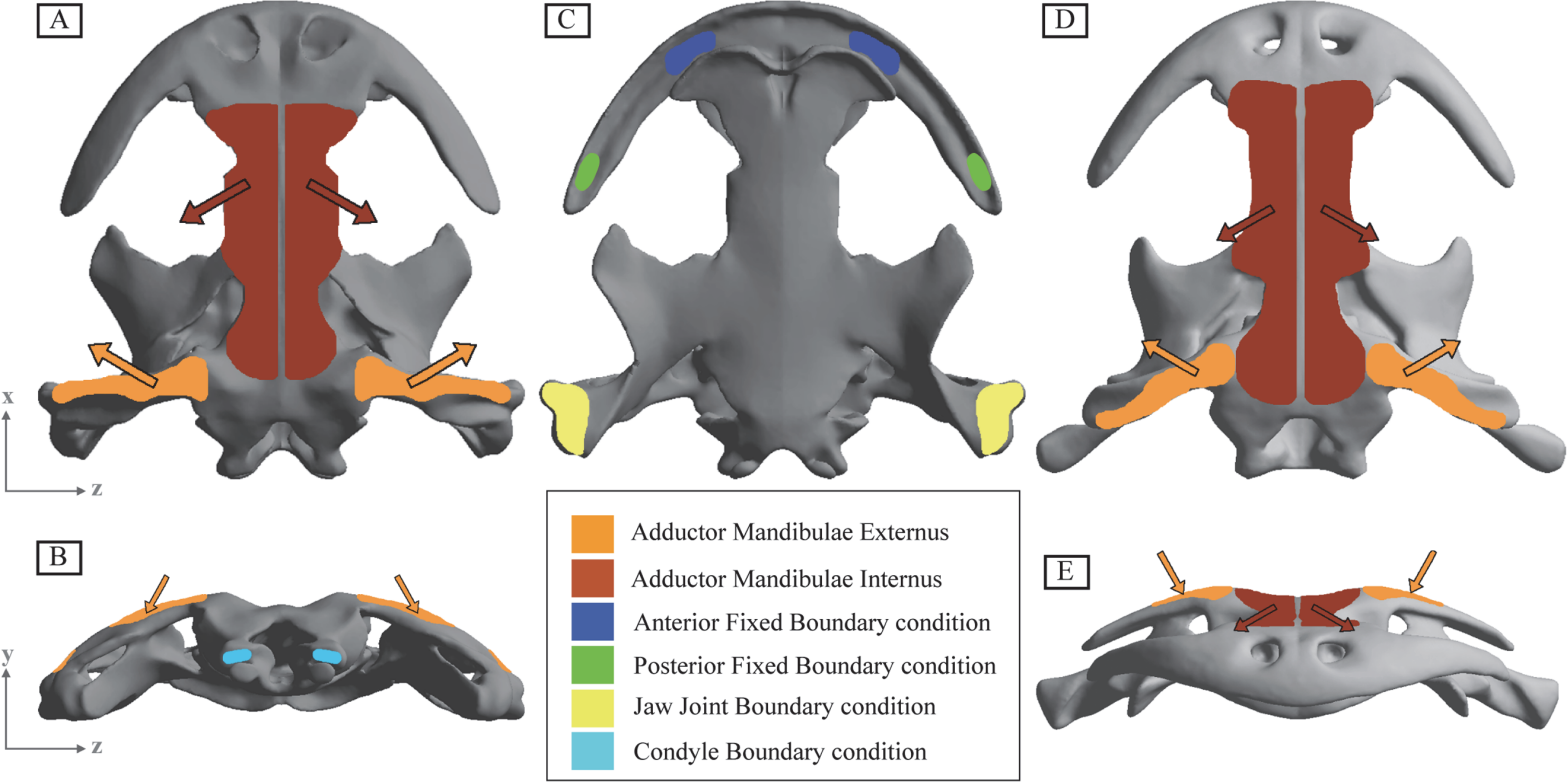
Boundary conditions of the two 3D modeled skulls of *Andrias davidianus*. A) Adult skull specimen in dorsal view. B) Adult skull specimen in occipital view. C) Subadult skull specimen in frontal view. D) Adult skull specimen in ventral view E) Subadult skull specimen in dorsal view.

Two loadings cases were analyzed involving bilateral and unilateral prehension (Fig. 1), considering the bilateral simulation when the two sides of the skull were biting while in the unilateral just the left side was biting, thus the right side was the balancing side.

For each biting case the analysis was repeated with two different positions of prehension: an anterior position, on the premaxilla-maxilla suture region, and a posterior prehension position on the most posterior part of the maxilla (most posterior teeth). To simulate the prehension a fixed boundary condition in the three dimensions was applied in these locations to model the moment that skull and mandibles contact the prey.

In all cases, the restrictions in displacements were applied at the jaw joint (the quadrate) in y-direction, where the skull is in contact with the jaw and at the double-headed occipital condyle to fix the skull in the x-direction related with the vertebral column (Fig. 1).

### Scaling the models

The adult and subadult specimens differ in shape and size. In order to compare the performance of structures that differ in shape and size and focus on how shape affects mechanical performance for a given loading condition, scaled values for the muscular contraction pressure need to be assumed. Some recent works have discussed how to scale the models to the same size in order to study the stress patterns or the strain energy [38, 39], thus proposing that size could be removed either by modifying the dimensions of the model or the values of the muscular forces or muscular pressures applied. Some criticisms have been made in recent papers [40] about the scale methodology proposed by [39], improving the 3D complex biological structures, especially regarding the complex inner cavities present in skulls.

**Fig 2:**
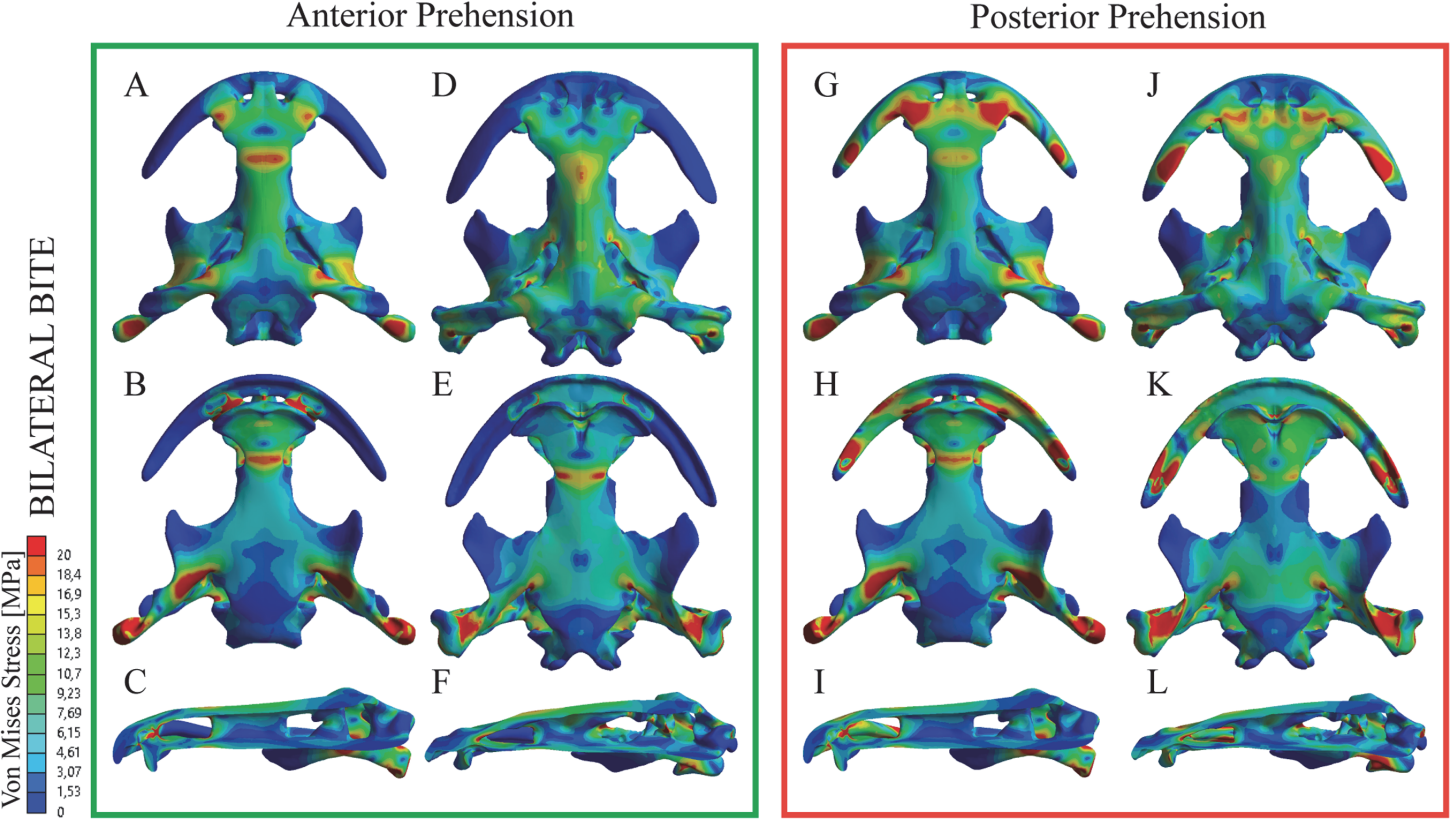
Von Mises Stress results in MPa of Bilateral loading under an anterior (A-F) or a posterior prehension (G-L) and a gape angle of 6°. Subadult specimen under the anterior prehension (A-C) and posterior prehension (G-I). Adult specimen under the anterior prehension (D-F) and posterior prehension (J-L). Dorsal views (A,D,G,J) and ventral views (B,E,H,K).

**Fig 3:**
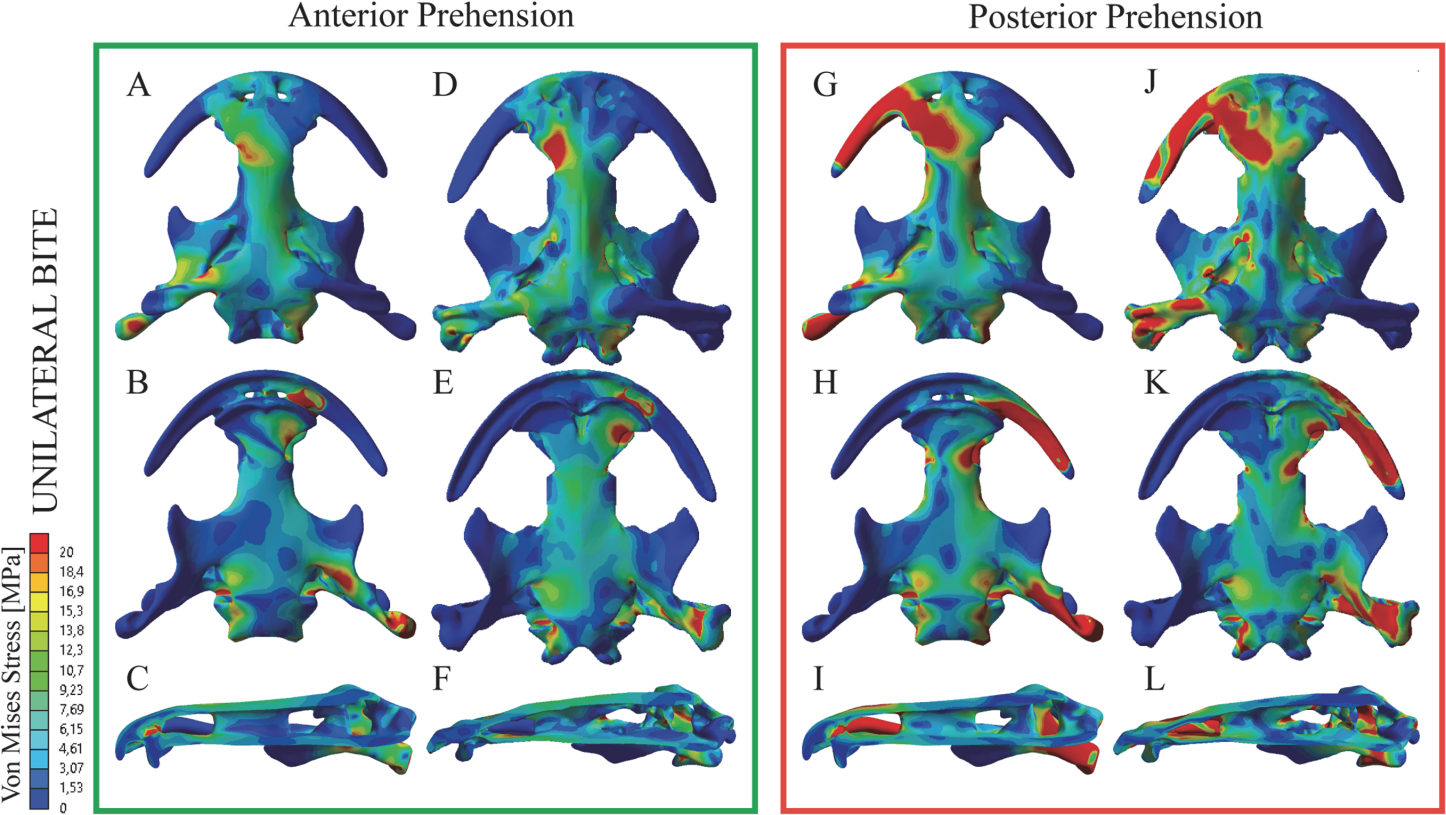
Von Mises Stress results in MPa of Unilteral loading (asymmetrical strike) under an anterior (A-F) or a posterior prehension (G-L) and a gape angle of 6º. Subadult specimen under the anterior prehension (A-C) and posterior prehension (G-I). Adult specimen under the anterior prehension (D-F) and posterior prehension (J-L). Dorsal views (A,D,G,J) and ventral views (B,E,H,K).

For scaling the models to allow comparison of the results obtained between them, the values for the adult model were used as the reference model, and the values applied in the subadult model were scaled from the reference one using two scaling methodologies: The first one following [38] and based in the differences of volume, and the second one following [39], see the results in SI, based in the differences of area. The values of the muscular pressure were scaled and the original size of the skulls was maintained to make comparisons. In addition, the muscular forces (F) were translated to pressures (P) by dividing their values by the surface area of muscular insertion (SM) where the force was applied. Considering the variables described, the two methodologies used were:

1. The forces of both models adult (A) and subadult (B) are related to the variation of the volume (V) of the skull, adapting the equations proposed by [38] to three-dimensional models (Figs. 2 and 3).

$$P_{A}=\frac{SM_{B}}{SM_{A}}{\left(\sqrt[{3}]{\frac{V_{A}}{V_{B}}}\right)}^{2}P_{B}$$ (Equation 1)

1. The forces of both models A and B are related to the variation of the surface (S) which surrounds the skull following the equations proposed in [39] (See S2 Fig. for the results using this methodology).

$$P_{A}=\frac{SM_{B}}{SM_{A}}\frac{S_{A}}{S_{B}}P_{B}$$ (Equation 2)

The formulation proposed in 1) is based in the mathematical demonstration that can be found in [38]. This formulation is used to scale the force values for plane models in bidimensional elasticity. When working in three-dimensional models, the models refer to homothetic transformations instead of quasi-homothetic transformations. Thus, the Jacobian matrix of a homothetic transformation *J*_*ij*_ is a diagonal matrix which describes a linear transformation to *J*_*11*_ = *J*_*22*_ = *J*_*33*_ maintaining the same proportionality in the three directions of the space, where α is the linear scaling constant in a homothetic transformation (Equation 3).

$$J_{ij}=\frac{\partial X_{ij}}{\partial x_{ij}}=\left[\begin{array}{l} \alpha \\ \;\alpha \\ \;\alpha \end{array}\right]$$ (Equation 3)

If the volume of model A can be written, *V*_*A*_ = *abc* from the Jacobian matrix of an homothetic transformation in a three-dimensional model, the volume of model B is *V*_*B*_ = *αaαbαc*. This relationship implies$\alpha =\sqrt[{3}]{\frac{V_{B}}{V_{A}}}$.

In this study, the interest of the comparison between the adult and subadult models lies in the Von Mises stress distribution. The Von Mises criterion is the most used criterion for predicting the yield of ductile materials. Bone can be assumed brittle [41] or ductile [39] but, according to [41], when isotropic material properties are used in cortical bone, the Von Mises criterion is the most accurate for predicting fracture location. As discussed in [38], in order to analyse the influence of the shape in front of the strength (related with stresses), Equation 4) need to be fulfilled to hold the same stress distribution between models when a homothetic transformation is done between two different models A and B.

$$\sigma_{A}\left(X_{1},X_{2},X_{3}\right)=\sigma_{B}\left(x_{1},x_{2},x_{3}\right)$$ (Equation 4)

Following [38], the relationship between the forces applied in model A and B is obtained from the relationship between equations in (5) where, by definition, the resultant of the stress distribution in the area where they are located is a resulting force.

$$F_{A}=\iint_{A_{A}}\sigma_{A}\left(X_{i}\right)dA=\iint_{A_{A}}\sigma_{A}\left(X_{i}\right)dLdT=\iint_{A_{B}}\sigma_{B}\left(x_{j}\right)\frac{1}{\alpha^{2}}dl=\frac{1}{\alpha^{2}}\iint_{A_{B}}\sigma_{B}\left(x_{j}\right)dldt=\frac{F_{B}}{\alpha^{2}}$$ (Equation 5)

According to the relationship between volumes obtained above, the relationship between F_A_ and F_B_ is stated in Equation 6. In this manner, both models were scaled with respect to volume, and using only the force values, allowing the comparison between them.

$$F_{A}={\left(\sqrt[{3}]{\frac{V_{A}}{V_{B}}}\right)}^{2}F_{B}$$ (Equation 6)

Equation 6 relates the volume of each specimen with the muscular force applied and agrees with the 2/3 power relationship based in allometric proportions, used in works as [42] between muscular forces and body mass in homogeneous materials and demonstrated in [43] and [44] for different groups.

## Results

Suture morphology is described on the basis of the CT scans with the aim to understand its mechanical significance and the potential intracranial flexibility of the skulls.

Regarding the FE Analysis, distribution and values of equivalent Von Mises stresses are recorded for the adult and subadult skulls in order to compare their behaviour under the effect of the loads and constraints of the bilateral and unilateral cases. Differences in the distribution of stress patterns considering the two gape angles analyzed are negligible and described together.

Furthermore, the estimated bite force that acts on the prey when the models are biting is recorded for the unilateral and bilateral cases as the reaction that appears in the fixed boundary condition that simulates the prehension for the two gape angles analyzed (Table 3).

**Table 3:** Loading cases analysed on the present study and estimated bite forces.

**Case**	**Specimen**	**Loading**	**Prehension position**	**Scaled**	**Bite force (gape 6°)**	**Bite force (gape 21°)**
A	Adult *A*. *davidianus*	bilateral	anterior	no	254.31 N	289.6 N
B	Adult *A*. *davidianus*	bilateral	posterior	no	278.49 N	146.71 N
C	Adult *A*. *davidianus*	unilateral	anterior	no	906.41 N	585.39 N
D	Adult *A*. *davidianus*	unilateral	posterior	no	582.76 N	575.38 N
E	Subadult *A*. *davidianus*	bilateral	anterior	yes	91.08 N	125.06 N
F	Subadult *A*. *davidianus*	bilateral	posterior	yes	105.2 N	127.61 N
G	Subadult *A*. *davidianus*	unilateral	anterior	yes	294.44 N	234.61 N
H	Subadult *A*. *davidianus*	unilateral	posterior	yes	199.8 N	202.38 N

### Suture morphology

**Fig 4:**
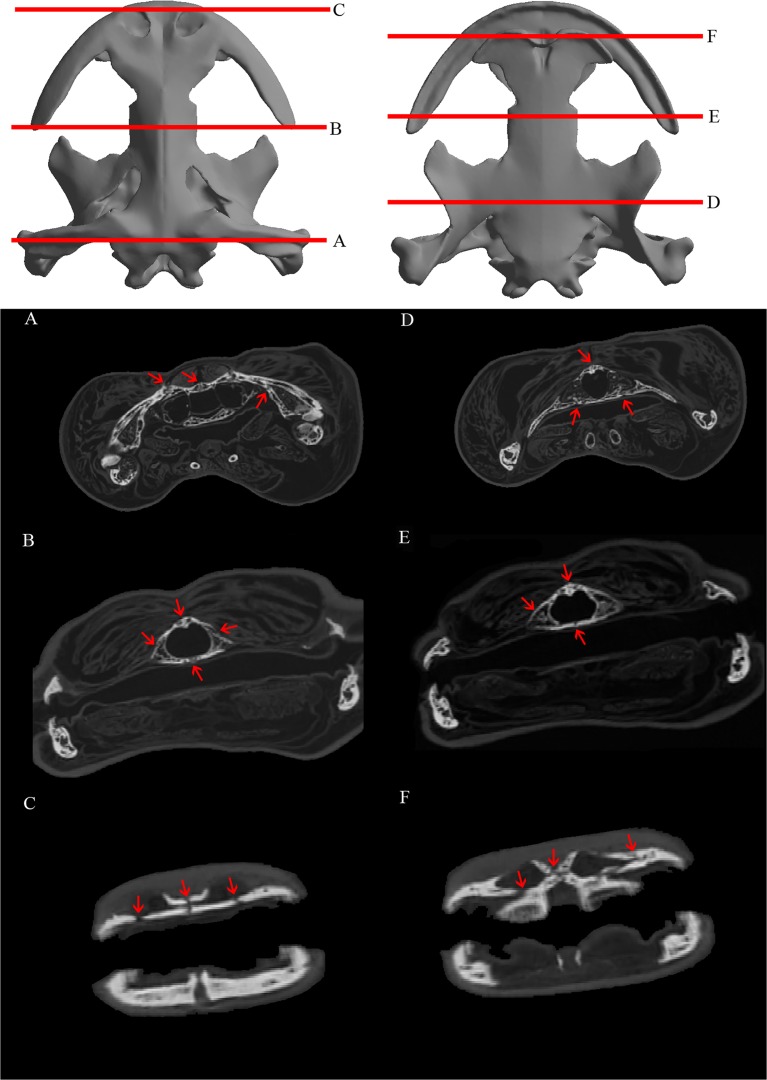
Cranial suture morphology of an adult specimen of the Chinese giant salamander (*Andrias davidianus*). CT slices along the skull with its correspondence to three-dimensional render. Red arrows indicate apparent sutures present.

**Fig 5:**
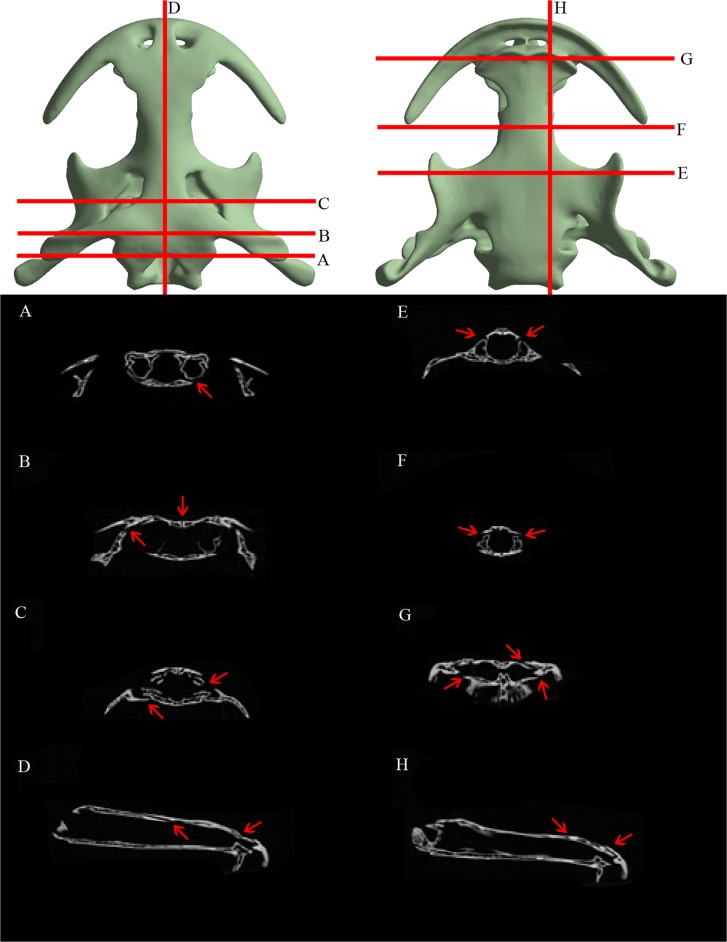
Cranial suture morphology of a subadult specimen of the Chinese giant salamander (*Andrias davidianus*). CT slices along the skull with its correspondence to three-dimensional render. Red arrows indicate apparent sutures present.

The CT scans provide information about suture morphology of the two specimens analyzed and reveal some differences between the specimens due to the different ontogenetical stage (Figs. 4 and 5). Different suture types can be discerned and assigned following the framework of [45].

The adult specimen (Fig. 4), from a posterior to anterior position, shows a groove suture type between the parietal and the squamosal and a vertical wall or steep bevel along the parietals suture that is not discernible in some areas, possibly fused. The suture is also apparent between the posterior ramus of the pterygoid and the squamosal, while the suture between the pterygoid and the parasphenoid is possibly grooved. On the other hand, on the fronto-parietal area there are vertical walls, slightly sloped (and fused in some points) between the frontals and parietals, but also a shelf suture type between the skull roof and the palate bones (parasphenoid-vomer). It’s also observable the inner cavities in the cross section of the skull roof-palate. Regarding the connection between the parasphenoid and the vomer, there is a vertical wall suture type between them. On the anterior part of the skull, the adult specimen has some apparent shelf type and vertical walls sutures between the skull roof bones, and in particular well developed between the maxilla-premaxilla, between the two sides of the premaxilla, the premaxilla-vomer, premaxilla-nasals, nasal-frontal and between the nasals. The subadult specimen (Fig. 5) has the same suture types as the adult specimen but few remarks should be added for comparison between the two specimens. Firstly, sutures are in most cases more apparent and discernible. This is especially the case of the vertical wall on the interparietal suture and between the skull roof bones (fronto-parietal) and the palate bones (parasphenoid-vomer). Interestingly, the frontal-parietal suture is clearly visible with a shelf suture type, not discernible in the adult specimen.

### Bilateral loading

Under a bilateral and anterior prehension, the adult skull results in maximum stress on the anterior position (left and right premaxilla-maxilla suture region) (Fig. 2 and supplementary videos) and the squamosal. There is a relatively large stress concentration on the skull roof: the fronto-parietal bones present an enlarged stress area, especially on the fronto-parietal suture region and at the nasal-frontal suture region. This latter stress region forms a belt surrounding the palate and following the vomer-parasphenoid suture. Considering the rest of the skull roof, the stress is present, but relatively less on the preorbital region, while the postorbital region shows stress areas around the posterior part of the squamosal with the exoccipitals bones without any measurable stress. From the palate, the vomer and the premaxilla present low levels of stress, and without any indication of stress on the maxilla. The central and posterior part of the parasphenoid shows low levels of stress, while the pterygoid has moderate and high levels of stress on the posterior ramus The subadult specimen, under the same equivalent scaled load (the values of the loads are different but scaled using the volume scaling presented above), present slightly higher stress levelsthan the adult specimen (Fig. 2). The stress pattern is similar, showing a stress belt around the prefrontal-nasal-frontal and vomer-parasphenoid suture regions. As in the adult specimen, the central part of the parietal is stressed, being similar to the stress found on the nasal region. The posterior part of the parietal shows low levels of stress while stress is higher on the posterior part of the pterygoid and the anterior part of the squamosal. On the palate, moderate levels of stress are also present on the vomer and premaxilla and the anterior part of the parasphenoid, while the maxilla show no stress. Pterygoid has low levels of stress on the anterior ramus increasing on the center of the bone and especially on the posterior ramus, contacting with the quadrate.

When the prehension is placed on a posterior position (Fig. 2 and S1 and S2 videos), on the most posterior part of the maxilla, the stress pattern is different in both adult and subadult skull models. On this loading, the stress peaks all the anterior portion of the prefrontal-nasal-frontal (skull roof) and vomer-parasphenoid (palate) sutures. In particular, high stress is present around the posterior part of each maxilla, most of the nasal region and the vomer, increasing on the region nasal-frontal, vomer-parasphenoid suture regions. Most of the parietal bone undergoes less stress with moderate levels on the posterior part of the squamosal. Considering the palate, as previously mentioned, the vomer, premaxilla and maxilla reveals relatively moderate and high levels of stress during posterior prehension, while the parasphenoid and exoccipital shows moderate and low levels of stress respectively while the anterior ramus of the pterygoids shows no sign of stress, increasing to the posterior ramus.

The subadult specimen also reveals similar stress patterns under the same load (Fig. 2) being slightly higher levels than the adult specimen. As in the adult specimen, the stress is mainly focused on the preorbital region: nasals, premaxilla, maxillas and the vomer. On the skull roof, the parietal and exoccipital shows low level of stress and similar to the levels found on the parasphenoid.

### Unilateral loading-Asymmetrical strike

Under a unilateral prehension on the left premaxilla-maxilla suture region (anterior position), the adult skull of *A*. *davidianus* shows maximum stresses similar to the results of the bilateral prehension on the anterior position (Fig. 3 and S3 and S4 videos). The stress peaks around the prefrontal-nasal-frontal sutures and the anterior part of the nasals. Moderate stress levels are also found along the parietal and squamosal, as well as the frontals. Regarding the palate, a peak of stress is present around the vomer-premaxilla suture, being present but in less proportion on the vomerparasphenoid and posterior part of the parasphenoid and the posterior part of the squamosal.

Under the same equivalent scaled loading conditions, the subadult skull model presents a stress pattern similar to the adult one (Fig. 3). As in the adult case, the stress peaks on the skull roof around the nasal region and specially the nasal-frontal suture. The frontals and most of the parietal and squamosal also show moderate levels of stress. Regarding the palate, the stress is present around the right vomer-premaxilla suture and the vomer-parasphenoid and the posterior ramus of the pterygoid.

On the adult skull model, when the prehension is located on the left posterior most position of the maxilla, the stress is concentrated on the preorbital region (Fig. 3 and S3 and S4 videos). In particular, the nasal region, the premaxilla, maxillas and the right squamosal shows important stress while the frontals, parietals and the left exoccipitals show low levels of stress. From the palate, stress peaks around the right portion of the vomer-parasphenoid suture, the posterior part of the maxilla and the posterior ramus of the pterygoid.

The stress pattern on the subadult skull model under the same equivalent scaled load is very similar (Fig. 3). The stress peaks around the left nasals, left posterior most maxilla and nasal-frontal suture. On the posterior part of the subadult skull, the right exoccipital and the posterior part of the squamosal presents stress areas. On the palate, the stress is especially focused on the right vomer-parasphenoid suture and posterior ramus of the pterygoid, with moderate levels of stress on the most posterior part of the parasphenoid.

## Discussion

Jaw prehension (i.e. direct bites: capturing prey by the jaws) and “secondary” bites, also called “manipulation bites” (i.e. holding, subduing or crushing prey after initial capture), are essential feeding mechanisms in extant amphibians and are performed both on land and in water [5,11]. Capturing prey by direct bites has also been suggested to represent the ancestral terrestrial prey capture mode in early tetrapods [4] that evolved from aquatic feeding modes and accordingly, it presented the primary capture mode in different groups of early amphibians [16–17, 46]. To improve grip when capturing prey by the jaws, some groups of early amphibians had a well-developed labyrinth tooth structure, with different teeth rows on the skull and jaws and, in some groups, fangs that probably further helped to catch and kill the prey. Accordingly, form and function of the biting system not only determine the success of energy uptake in extant amphibians but also played an essential role during the evolution of tetrapods.

### Position of the prey

Our analysis reveals that the position where prey contacts the maxilla-premaxilla is crucial. Although teeth are present along the maxilla and the premaxilla, the stress distribution is clearly different when biting occurs on the premaxilla or at the posteriormost part of the maxilla. When the prehension is positioned on the premaxilla-maxilla suture the stress is probably dissipated and absorbed due to the key role of two cranial sutures: the nasal-frontal and the vomer-parasphenoid sutures, and in lesser way, the fronto-parietal suture. Sutures play an important role for the stability of the bones for the strength, torsional and compression forces present in the skull during different activities [47–49]. In *Andrias* the connections of neighboring bone elements in the snout region (nasal, frontal, premaxilla, maxilla, vomer) are of special interest because of the presence of shelf type sutures (overlapping bones) that most probably play a key role in dissipating and absorbing the stress during feeding. Only the frontal-parietal suture is not a shelf suture, showing a slight slope and fusion in some regions of the adult specimen, possibly indicating that this suture plays a minor role in dissipating the stress in comparison with shelf suture type.

On the other hand, a posteriormost prehension causes high stress levels among most of the maxilla, nasals, vomers and frontals and to a lesser extent on the frontals and parietals. In a posterior bite, most of the stress may be absorbed by the same sutures described above though the stress present in most of the maxilla might be difficult to dissipate. Nevertheless, in both cases, the stress pattern is confined especially to the preorbital region, while the braincase exhibits very low or no levels of stress even though the prehension is placed on an anterior or posterior position. However, a direct comparison between an anterior and a posterior bite shows that the posterior bite is probably less optimal as the skull is exposed to higher stress levels. In the same way, bite force usually increases posteriorly in a bilateral bite when the gape angle is 6°, but decreases posteriorly in an asymmetrical bite (Table 3). The increased stresses in the posterior part of the skull might be consequence of an increased torsion because the posterior bite is on such a wide part of the skull and perhaps the majority of the muscle forces act medio-laterally, rather than dorso-ventrally, hence reducing the reaction force of the resultant vector in the y direction. More importantly, this stress pattern is most probably related to the lack of a bony bridge between the posterior end of the maxilla and the anterior quadrato-squamosal region, resulting in the suspension of the posterior maxilla, resulting in extended areas of stress during feeding in comparison with an anterior bite. This arrangement is atypical for forceful biters amongst vertebrates who show the highest bite forces in the posterior jaw, close to the jaw joint where the out lever is shortest [12]. The hind region of the skull-jaw-apparatus is in fact used by many vertebrates to process, cut and crash their food [12, 50–51]. Extant salamanders however, lack a stabilizing bony bridge between maxilla and squamosal/quadrate (with only the exception of *Echinotriton* (2 spp.) and *Tylototriton* (ca. 20 spp.) and very few specialized salamanders (e.g. amphiumids and desmognathid plethodonthids) are in fact known to be forceful biters [5], compared to other vertebrate taxa. The absence of the bony bridge could be related to the evolutionary history of early urodels as revealed by functional implications on the bone ossification and adductor insertion from the paleontological record [52]. The giant salamanders, however, are known to prey on elusive, struggling, and hard prey (such as crayfish, fish, other amphibians or small mammals) that needs to be fixed, subdued and eventually crushed before swallowing [5, 35]. Accordingly, the capability to exert a forceful bite may increase performance in handling such prey but contrary to most other vertebrates, the highest forces might be applied in the anterior mouth region where mechanical stress is much lower during a bite compared to the posterior jaw-region. Given general similarities of the skull bone architecture in *Andrias* and many other metamorphosed urodeles as salamandrids and ambystomids (e.g. large orbits, absence of bony contact between pteygoid and maxilla, extensive interpterygoid vacuities and pedicellate teeth) [21,34], the combination of these osteological characters is probably reflects the general functional scheme, and consequently the stress pattern, found in other urodele groups.

### Asymmetrical strikes

Giant salamanders are capable of highly asymmetrical strikes—a unique feature amongst vertebrates. Asymmetrical suction strikes and induced asymmetrical bites in *Cryptobranchus alleganiensis* and *Andrias japonicus* have been described [35, 53] and showed that giant salamanders can direct their strike to one side by using unilateral jaw- and hyobranchial movements. This behavior is probably induced by bilaterally asynchronous motor patterns and facilitated by the strongly curved mandibles connected by a flexible symphysis [5]. Asymmetrical strikes are unusual and specialized features in giant salamanders but might indeed be beneficial in such sit-and-wait or ambush-predators to capture laterally approaching prey. However, once captured by an asymmetrical strike, large, elusive and struggling prey have to be brought to the anterior jaw region to be subdued by a strong bite as the posterior jaw-skull apparatus is not optimal to handle high stress levels induced by strong bites. In addition, if the stress values were to approach failure, this region would fail first, as shown in our models.

### Growth changes

The two skull models analyzed vary in 50% of total length, and show slight morphological differences. Although this study is only based on two individuals, the digital modeling reveals that through the ontogeny of *A*. *davidianus* the feeding system experienced changes as shown by the results. The analyses performed revealed that maximum stress levels are lower on the adult specimen compared to subadult under equivalent scaled loadings. The general stress pattern in both skull models are similar enforcing the importance of the naso-frontal and vomer-parasphenoid regions to dissipate, redistribute and/or absorb the stress and energy due to the prehension, specially on the subadult specimen, where the stress levels are clearly low or nonexistent on the parietals and braincase region. The suture morphology between the two specimens also reveals that some sutures, especially from the skull roof, fuse during the ontogeny. This is the case of the suture between the parietals and between the frontal and parietal. These sutures are well discernible in the subadult specimen, but mostly fused in the adult stage. Thus, our results suggest that during ontogeny of *A*. *davidianus*, the morphological changes of the skull result in lower stress levels during bites. On the other hand, some sutures from the vomer-parasphenoid region and the snout (premaxilla-maxilla-nasal-frontal) were not fused in the adult animal, providing further support that they play a key role on the absorption and dissipation of the stress during a bite.

### Evolutionary implications

Digital modeling implies assumptions (i.e. skull morphology smoothing, myological simplifications) to obtain valuable data about functional morphology of living and related extinct taxa. This is especially relevant in reconstructing musculoskeletal anatomy in extinct taxa, where more data are missing [54]. For this reason, the analysis of living taxa is of special interest as important paleobiological implications can be inferred for extinct taxa. The results herein found in the skull models of *A*. *davidianus* about feeding mechanics offer a new window to infer the paleobiology of lissamphibians (including basal salamanders) and complements a biomechanical approach using in vivo data and CFD simulations [7]. *Andrias* has been used to infer the paleobiology of different groups of early tetrapods such as temnospondyls due to the gigantic size of some of its members [55]. Of special interest is the morphology and development of the musculature. The reconstruction of soft tissues in fossil taxa is mainly based on the attachments of the musculature preserved in bones and inferred by a phylogenetic approach using the extant phylogenetic bracket [56]. In the case of early tetrapods and early amphibians, basal salamanders, some studies attempted to reconstruct the cranial and hyobranchial musculature [32, 57].

#### Evolution of cranial form and function

Caudate and urodele cranial evolution is probably related with functional changes associated with the loss of most circumorbital bones (with the exception of the prefrontal and lacrimal) and bone loss at the posterior portion of the skull at the tabular, supratemporal, intertemporal and postparietal and the dissaparence of a squamosal notch [21]. Of special interest is that these bones (particularly postfrontal, postorbital and jugal) failed to ossify, allowing the expansion of the musculature adductor (particularly, the AMI) to the dorsal surface of the frontal and the parietal [52]. These bone losses may explain the evolution of the stress patterns in salamander evolution from dissorophoid amphibians to most basal extant groups of salamanders as Cryptobranchoids. The paleontological evidence reveals that amphibamids and branchiosaurids still retained circumorbital bones (as postfrontal, postorbital and jugal), although poorly developed and possibly ossified late in ontogeny, and maintained a poorly developed bony bridge between the maxilla and the quadrato-squamosal region [52, 58]. This cranial configuration probably resulted in a myological development similar to basal extant salamanders, with an increasing development of AMI in the dorsal part of the frontals and parietals and consequently the stress patterns was not equal, but probably presented some similarities as happens in basal extant salamanders as *Andrias*. This scheme continue evolving and the Middle Jurassic Karaurids represents an important step for the stress distribution in the salamander lineage due to the loss of several circumorbital and posterior skull table bones (caused by the failure of bone ossification or bone fusion) [52, 59]. These changes caused the disappearance of a bony bridge between the maxilla and the quadrate-squamosal-jugal region. In general terms, the cranial scheme of karaurids possibly indicate that stress pattern and distribution between karaurids and cryptobranchids had significant similarities.

#### Adductor musculature evolution

Adductor muscles externus and internus (AME and AMI respectively) are key to understanding the feeding mechanics of extant and extinct anamniotes during jaw prehension. The insertion and development of these muscles on the different groups of early tetrapods and amphibians was probably diverse possibly causing different stress patterns on the skull under different feeding regimes. In the skull of *A*. *davidianus*, AME is placed on the dorsal part of the squamosal, while AMI acts as antagonist of the Depressor Mandibulae (DM) muscle and has a larger insertion area than AME, occupying most of the parietal, frontal and nasal bones of the skull roof. This myological scheme is possibly different from lepospondyls (lysorophians in particular) because while AME and AMI are also placed in dorsal part, but AME was probably the most developed adductor muscle in comparison with poorly developed AMI. This scheme probably reflects a different feeding system and lifestyle, as potential burrower animals [60]. On the contrary, the myological scheme present in *Andrias* was possibly similar tosome groups of early tetrapods and amphibians, including for example, the karaurids, the Temnospondyli group of Dissorophoidea (e.g.branchiosaurids and dissorophids), but probably not for other groups such as Stereospondyls or plagiosaurids where insertion was ventral instead of dorsal as present in extant salamanders as well as the presence of a developed adductor mandibulae posterior (AMP) [32, 57]. These myological differences added to the important morphological and structural differences present in these temnospondyl groups suggests that *A*. *davidianus* is not a good model to infer the paleobiology of some groups as stereospondyls or plagiosaurids. However, groups such as dissorophoids possibly presented a very similar myological and structural pattern as present in giant salamanders, revealing that probably these groups used suction and jaw prehension feedings. Dissorophoids are of special interest because they are central actors of the lissamphibian origin debate [61]. Different radiations of dissorophoids, in particular the amphibamids have been hypothesized as potential ancestors of the lissamphibians. As discussed above, the cranial evolution in form and function indicates that in dissorophoids (particularly amphibamids) AMI was increasingly developed in the dorsal part of the frontals and parietals, being well developed in karaurids.

### Limitations of the models

The FE models of adult and subadult stages of *Andrias davidianus* represents the first study that attempts to reconstruct the 3D stress patterns on the skull during a bite of an anamniote. Our FE amphibian skull models open the door to future simulation in different salamander taxa to expand the results reported to other amphibian clades and explore the evolutionary history of urodeles. The models are based on detailed geometry and information about musculature, particularly adductor mandibulae. However, any modeling implies an approximation of the reality based on different assumptions. These assumptions should be considered for the interpretation of the results and more importantly, for future improvements of the models. The analyses here reported simulate two different feeding behaviors and assume that all muscles work simultaneously and maximally; however living giant salamanders load their skulls and mandibles in different ways, not validated here and that could represent more complicated loadings, for example considering different gape angles. It should be also noted that there is a gap of information about cranial material properties of amphibians. This information, together with anisotropy and orthotropic data will allow us to refine and better understand the stress patterns of the model input. Moreover, experimental data should also include information about bite forces, cranial muscle architecture and cranial contractile properties (e.g. muscle fiber length, PCSA, pinnation angle, sarcomere length etc) to validate the FE models. Future analyses will incorporate the mandible and perform dynamic analysis (as MDA) to deepen on the functional implications of the asymmetrical strikes on the mandible. Moreover, material properties of the pedicellate teeth, hyobranchial skeleton as well as to model sutures could help to refine the models. Finally, to validate the output model future analyses would need to include strain or displacement data from strain gauges, speckle interferometry or XROMM.

## Conclusions

The 3D bite modeling of *Andrias davidianus* provides new insights on the feeding ecology of giant salamanders. For elusive or large prey these animals use a biting mechanism where the optimal prehension position for the structural performance of the skull is on the premaxilla-anterior part of the maxilla rather than nearing the posterior part of the maxilla. This fact is probably related to the lack of a bony bridge between the posterior end of the maxilla and the anterior quadrato-squamosal region. Otherwise, giant salamanders performed asymmetrical strikes that may be beneficial for a sit-and-wait predator to laterally capture prey but that once captured is probably immobilized by a symmetrical bite. Cryptobranchids are of special interest from an evolutionary point of view as they share many ancestral characteristics that may help to deduce the function of early lissamphibian feeding systems with analogous myoskeletal cranio-cervical constellations.

## Supporting Information

S1 Fig Meshed models with an adaptive mesh of hexahedral elements. Subadult skull specimen in dorsal (A) and ventral (B) views and adult skull specimen in dorsal (C) and ventral views (D).
(TIF)

S2 Fig Von Mises Stress results in MPa of Surface scaled models under a bilateral and unilateral loadings of the subadult skull specimen. For bilateral bite: anterior prehension, A) dorsal B) ventral and C) mid-line section views. Posterior prehension, D) dorsal E) ventral and F) mid-line section views. For unilateral bite: anterior prehension, G) dorsal H) ventral and I) mid-line section views. Posterior prehension, J) dorsal K) ventral and L) mid-line section views.
(TIF)

S1 Video Bilateral loading under an anterior prehension in the adult specimen of of the Chinese giant salamander (*Andrias davidianus*). Von Mises Stress results in MPa.
(AVI)

S2 Video Bilateral loading under a posterior prehension in the adult specimen of of the Chinese giant salamander (*Andrias davidianus*). Von Mises Stress results in MPa.
(AVI)

S3 Video Unilateral loading under an anterior prehension in the adult specimen of of the Chinese giant salamander (*Andrias davidianus*). Von Mises Stress results in MPa.
(AVI)

S4 Video Unilateral loading under a posterior prehension in the adult specimen of of the Chinese giant salamander (*Andrias davidianus*). Von Mises Stress results in MPa.
(AVI)
