# Supplementary figures and images for: 3D Bite Modeling and Feeding Mechanics of the Largest Living Amphibian, the Chinese Giant Salamander Andrias davidianus (Amphibia:Urodela)
Source: PLoS One. 2015 Apr 8;10(4):e0121885. doi: 10.1371/journal.pone.0121885 (PMC4390218; doi:10.1371/journal.pone.0121885)

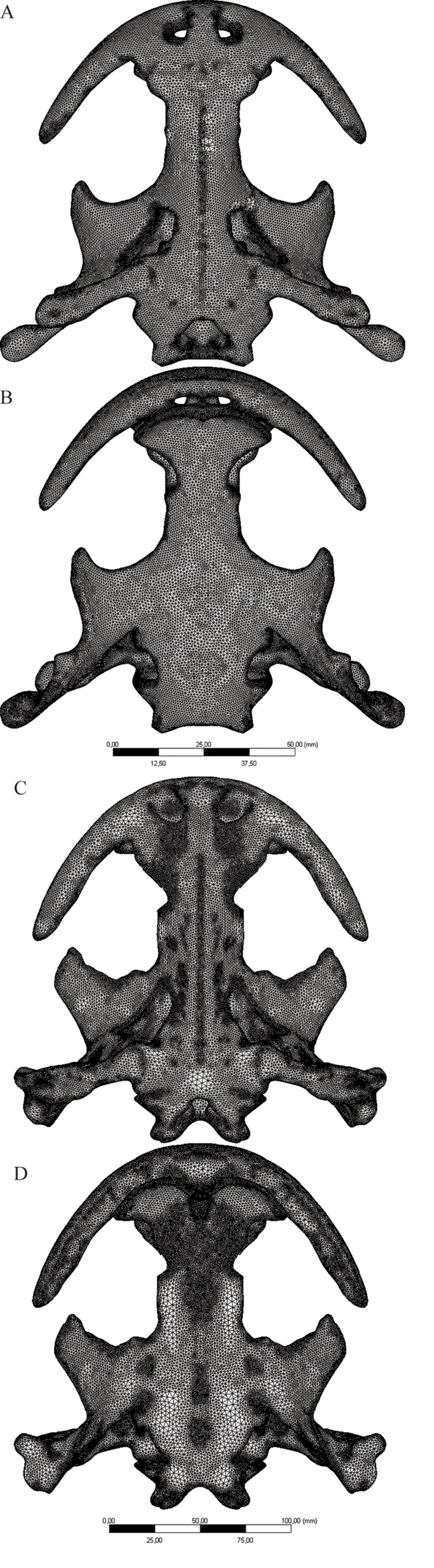

Supplement: S1 Fig — Subadult skull specimen in dorsal (A) and ventral (B) views and adult skull specimen in dorsal (C) and ventral views (D). (TIF) [file pone.0121885.s001.tif]

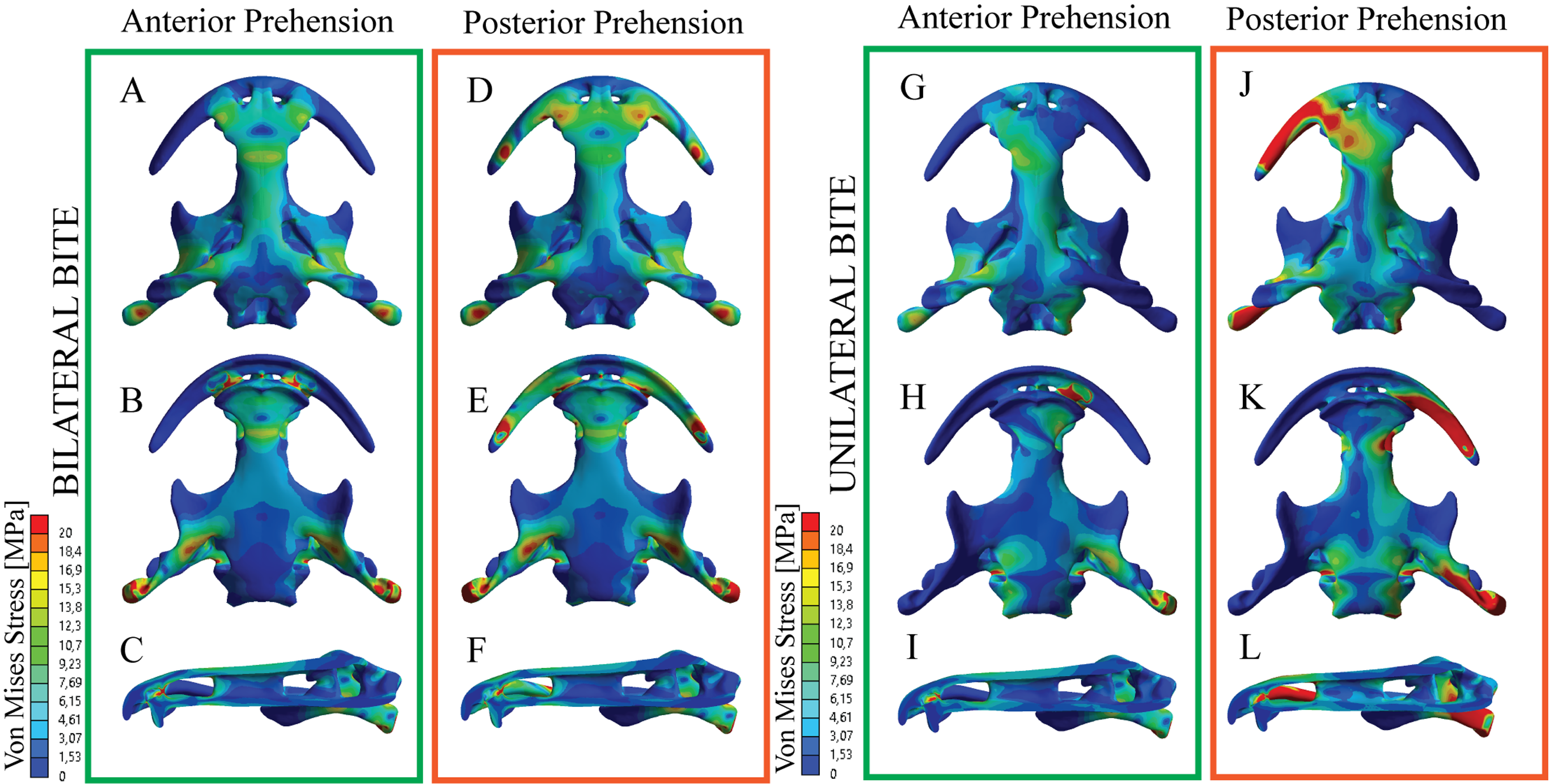

Supplement: S2 Fig — For bilateral bite: anterior prehension, A) dorsal B) ventral and C) mid-line section views. Posterior prehension, D) dorsal E) ventral and F) mid-line section views. For unilateral bite: anterior prehension, G) dorsal H) ventral and I) mid-line section views. Posterior prehension, J) dorsal K) ventral and L) mid-line section views. (TIF) [file pone.0121885.s002.tif]
